# Comparing Large Language Models and Human Programmers for Generating Programming Code

**DOI:** 10.1002/advs.202412279

**Published:** 2024-12-30

**Authors:** Wenpin Hou, Zhicheng Ji

**Affiliations:** ^1^ Department of Biostatistics Mailman School of Public Health Columbia University New York City NY 10032 USA; ^2^ Department of Biostatistics and Bioinformatics Duke University School of Medicine Durham NC 07024 USA

**Keywords:** artificial intelligence, computer programming, human‐computer interaction, large language models

## Abstract

The performance of seven large language models (LLMs) in generating programming code using various prompt strategies, programming languages, and task difficulties is systematically evaluated. GPT‐4 substantially outperforms other LLMs, including Gemini Ultra and Claude 2. The coding performance of GPT‐4 varies considerably with different prompt strategies. In most LeetCode and GeeksforGeeks coding contests evaluated in this study, GPT‐4, employing the optimal prompt strategy, outperforms 85 percent of human participants in a competitive environment, many of whom are students and professionals with moderate programming experience. GPT‐4 demonstrates strong capabilities in translating code between different programming languages and in learning from past errors. The computational efficiency of the code generated by GPT‐4 is comparable to that of human programmers. GPT‐4 is also capable of handling broader programming tasks, including front‐end design and database operations. These results suggest that GPT‐4 has the potential to serve as a reliable assistant in programming code generation and software development. A programming assistant is designed based on an optimal prompt strategy to facilitate the practical use of LLMs for programming.

## Graphical abstract

The performance of seven large language models is evaluated for programming code generation across various prompts, languages, and task difficulties. GPT‐4 consistently outperforms other models and excels in tasks such as code translation, error learning, and efficient code generation. It surpasses 85% of human participants in most LeetCode and GeeksforGeeks contests when using the optimal prompt strategy.

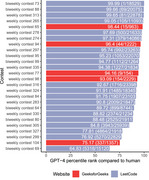

## 1 Introduction

The emergence and growth of large language models (LLMs), such as GPT‐4, Gemini, Claude, and Llama, are poised to revolutionize various sectors, including computer programming,^[^ ^1^, ^2^, ^3^, ^4^, ^5^, ^6^, ^7^, ^8^ ^]^ education,^[^ ^9^, ^10^ ^]^ and biomedical research.^[^ ^11^, ^12^, ^13^, ^14^, ^15^ ^]^ A notable feature of LLMs is their usability. They can be guided to accomplish various complex tasks through prompts, which are tailored natural language text directives specifying user expectations. This feature substantially increases the inclusivity in various domains, especially in computer programming. Although a foundational level of training and knowledge remains essential for utilizing and verifying code, LLMs could significantly lower the barriers for those without extensive backgrounds in computer science or programming. By democratizing access to computer programming, LLMs have the potential to expand and diversify the programming workforce.

The coding capabilities of LLMs have been benchmarked through two types of studies. The first type designed benchmark datasets, including HumanEval,^[^ ^16^ ^]^ MBPP,^[^ ^17^ ^]^ APPS,^[^ ^18^ ^]^ CoNaLA,^[^ ^19^ ^]^ and CodeContests,^[^ ^20^ ^]^ to evaluate the performance of LLMs. HumanEval is one of the most widely used benchmark datasets. On HumanEval, Claude 2^[^ ^21^ ^]^ and Gemini Ultra^[^ ^22^ ^]^ achieve the best performance, with accuracies of 71.2% and 74.4%, respectively. GPT‐4,^[^ ^23^ ^]^ Gemini Pro,^[^ ^22^ ^]^ and Code Llama^[^ ^24^ ^]^ exhibit comparable performance, with accuracies of 67.0%, 67.7%, and 67.0%, respectively. In contrast, GPT‐3.5^[^ ^23^ ^]^ and Llama 2^[^ ^25^ ^]^ perform worse, with accuracies of 48.1% and 29.9%, respectively. The second type of study assessed the performance of LLMs using programming tasks directly obtained from coding practice websites, such as LeetCode.^[^ ^1^, ^3^, ^8^, ^23^ ^]^ According to the GPT‐4 technical report by OpenAI,^[^ ^23^ ^]^ GPT‐4 achieved accuracies of 75.6%, 26.3%, and 6.7% on easy, medium, and hard LeetCode coding tasks, respectively. A more recent study^[^ ^1^ ^]^ reports that GPT‐4 reached one‐attempt accuracies of 68.2%, 40%, and 10.7%, and five‐attempt accuracies of 86.2%, 60.0%, and 14.3% on easy, medium, and hard tasks, respectively. In comparison, GPT‐3.5 achieved substantially lower accuracies of 58%, 18%, and 1% on easy, medium, and hard tasks, respectively.^[^ ^8^ ^]^

Several major limitations are evident in these studies. First, existing research primarily relies on a simplistic prompt strategy that involves repeatedly presenting the same programming task to LLMs, without exploring the potential of alternative prompt strategies. Previous studies have demonstrated that varying prompt strategies can substantially impact LLM performance,^[^ ^26^, ^27^ ^]^ suggesting that LLM programming capabilities could be further enhanced through prompt engineering. Second, there is a lack of rigorous comparison between the programming abilities of LLMs and humans. Such comparisons are crucial for understanding LLM limitations and identifying ways they can effectively collaborate with humans in development environments. While one study^[^ ^1^ ^]^ compares GPT‐4's performance to the acceptance rate of LeetCode programming tasks by humans, it notes that this rate includes all historical submissions, many of which may be copies of published solutions. Hence, the acceptance rate may not accurately represent the actual coding abilities of human programmers, leading to biased comparison results. Third, the focus of most studies has been exclusively on the Python programming language, leaving the performance of LLMs in other widely used languages, such as Java, Javascript, and C++, underexplored. Additionally, LLMs' capability to translate across different programming languages remains poorly understood. Code translation is a critical aspect of modern software development, as it enables interoperability between platforms, facilitates legacy system integration, and supports the migration of codebases to newer languages or frameworks. LLMs have the potential to streamline cross‐platform development and enhance productivity in diverse programming environments. Fourth, prior research has predominantly concentrated on the accuracy of code generated by LLMs, neglecting other important aspects such as code execution time and the ability of LLMs to learn from coding errors. These aspects are vital for the development of programming code and software.

In this study, we conducted a systematic assessment of the ability of seven LLMs to generate programming code. These included GPT‐4^[^ ^23^ ^]^ and GPT‐3.5,^[^ ^28^ ^]^ developed by OpenAI; Gemini Ultra^[^ ^22^ ^]^ and Gemini Pro,^[^ ^22^ ^]^ developed by Google; Claude 2,^[^ ^21^ ^]^ developed by Anthropic; and Llama 2^[^ ^25^ ^]^ and Code Llama,^[^ ^24^ ^]^ developed by Meta. We addressed the limitations identified in previous research by investigating how different prompt strategies affect LLMs' coding performance, comparing the coding abilities of LLMs with those of human programmers in a competitive setting, evaluating LLMs' performance in generating and translating code across various programming languages, and examining LLMs' computational efficiency and their ability to learn from past errors. We also evaluated GPT‐4's performance in areas such as front‐end design, the application of development frameworks, and database operations. We discovered that GPT‐4 is the most effective LLM in generating programming code, and that employing an optimal prompt strategy substantially enhances GPT‐4's coding abilities compared to the standard prompt strategy used in previous studies. With this optimal strategy, GPT‐4 outperforms 85% of human participants in most contests on LeetCode and GeeksforGeeks in a competitive environment, demonstrating its strong potential as a tool in programming code generation and software development.

## 2 Results

### 2.1 Primary Evaluation Datasets

In this study, we primarily evaluated LLMs using programming tasks sourced from LeetCode and GeeksforGeeks, the two most visited platforms for practicing programming (Figure S1, Supporting Information). We selected LeetCode as the primary source for our evaluation dataset, as its programming tasks can be directly compiled by copying and pasting contents from the LeetCode website. In contrast, GeeksforGeeks does not permit copy‐and‐paste operations, requiring manual transcription of the programming tasks. Consequently, only a small number of tasks were compiled from GeeksforGeeks. We did not include benchmark datasets such as HumanEval and MBPP, since the performance of human programmers on these datasets is unknown.

We manually compiled 100 programming tasks from 25 randomly selected LeetCode contests. Four of these contests were held before September 2021, the knowledge cutoff of GPT‐4 and GPT‐3.5. The remaining contests, representing various months and years, were held after September 2021 (Figure S2, Supporting Information). Each LeetCode contest comprises four programming tasks with easy, medium, or hard difficulties, as determined by LeetCode. A typical LeetCode task includes a problem description, example test cases with expected outputs and explanations, constraints, and a code template (Figure S3a–d, Supporting Information). LeetCode assesses the correctness of a solution by checking whether it generates accurate outputs for all test cases. If a solution fails to pass all test cases, LeetCode provides error messages (Figure S3e, Supporting Information). LeetCode accepts solutions in various programming languages. In this study, we evaluated GPT‐4's performance in generating code in Python3, C++, Java, and Javascript. For successful solutions, LeetCode reports the percentiles of memory usage and running time compared to all human submissions (Figure S3f, Supporting Information). These tasks encompass a broad spectrum of topics in algorithm design, and most topics are covered by multiple programming tasks. (Figure S4, Supporting Information).

We also manually compiled 15 programming tasks from five GeeksforGeeks contests, all of which were published after September 2021. The tasks from GeeksforGeeks have content similar to that of LeetCode tasks. Additionally, GeeksforGeeks provides error messages when a solution fails to pass all the test cases. Due to the limited number of programming tasks from GeeksforGeeks collected in this study, they were used solely for comparing contest performances between LLMs and humans.

### 2.2 Prompt Strategies

**Figure 1:**
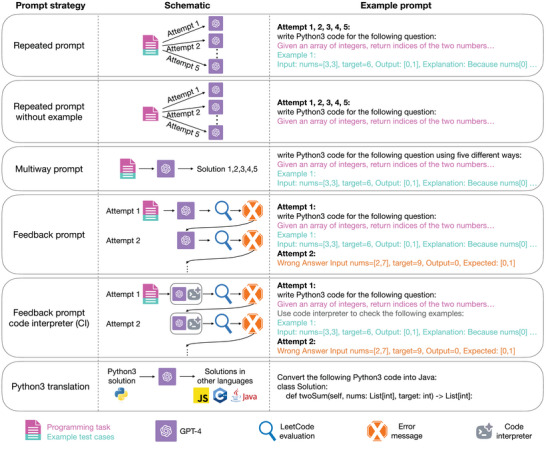
Schematics and example prompts illustrating the prompt strategies that were developed and assessed in this study.

We designed six prompt strategies to test the performance of LLMs in solving programming tasks (**Figure** **1**, Experimental Section), each chosen to evaluate a distinct aspect of LLM capabilities in line with known theoretical frameworks and practical considerations. In the first strategy (repeated prompt), the full programming task, along with an example test case, is repeatedly given to the LLM. This approach, commonly used in existing studies,^[^ ^16^ ^]^ evaluates the model's baseline ability to parse instructions and generate solutions without incorporating feedback or intermediate error signals. The second strategy (repeated prompt without example) is identical to the first but omits the example test case. This tests whether LLMs can still effectively solve problems without direct reasoning cues, inspired by the “chain‐of‐thought” concept,^[^ ^26^ ^]^ which suggests that including intermediate reasoning steps can improve performance. The third strategy (multiway prompt) asks the LLM to generate five different solutions simultaneously, leveraging its stochastic output generation to explore solution diversity and increasing the likelihood of success, a critical aspect of problem‐solving in programming tasks.^[^ ^20^ ^]^ The fourth strategy (feedback prompt) builds on real‐world debugging practices by introducing an iterative feedback loop. The LLM receives the full programming task, and its output is evaluated by LeetCode. If the solution fails, the model is given the error message as input to refine its solution, reflecting its ability to adapt and improve based on external signals. The fifth strategy (feedback CI prompt), is specifically designed for GPT‐4 and leverages a feedback‐driven approach grounded in principles of iterative refinement and tool augmentation. Code interpreter (CI) provides GPT‐4 with a Python working environment to execute and validate its code against example test cases. Through iterative cycles, GPT‐4 evaluates both newly failed test cases and previously identified ones, refining its solutions based on real‐time execution outputs and error signals. This approach evaluates GPT‐4's ability to effectively integrate external tools into its reasoning and learning processes. The sixth strategy (Python3 translation) is also specific to GPT‐4 and evaluates its ability to adapt solutions across programming languages. Python3 code generated using the feedback CI prompt strategy is translated into Java, JavaScript, and C++, testing the model's capacity to maintain solution logic while adapting to different syntaxes and paradigms.^[^ ^16^ ^]^

These strategies were selected to comprehensively assess LLM performance in key areas: instruction parsing, reasoning, solution diversity, feedback incorporation, external tool utilization, and cross‐language translation. Together, they provide a framework that mirrors real‐world programming challenges, ensuring the methods are systematic and grounded in both theoretical considerations and practical relevance.

### 2.3 Comparing Success Rates of GPT‐4 with Different Prompt Strategies

We first tested the performance of GPT‐4 with six prompt strategies on the 100 LeetCode programming tasks. Each prompt strategy had a maximum of five opportunities to solve each task. We recorded the success rates of each prompt strategy, both for solving the tasks on the first attempt (one‐attempt) and within five attempts (five‐attempt).

We compared GPT‐4's five‐attempt success rate for tasks published before or after its knowledge cutoff. GPT‐4 successfully solves all programming tasks published before September 2021 within five attempts, whereas the success rate substantially drops for tasks published after September 2021 (Figure S5, Supporting Information). A potential reason is that tasks before September 2021 were included as part of GPT‐4's training data. This results in data leakage, where information unavailable at prediction time is included during the model training process. Consequently, the performance on tasks published before September 2021 may not accurately reflect GPT‐4's ability to handle new programming tasks not seen in its training data, an occurrence that is highly likely in real‐world practice. To more rigorously assess GPT‐4's coding performance and to ensure a more fair comparison with other LLMs, our subsequent analyses exclusively focus on contests and tasks published after September 2021.

**Figure 2:**
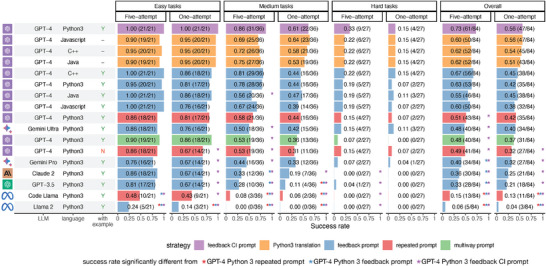
Five‐attempt and one‐attempt success rates for tasks with varying difficulty levels. Each row represents a different prompt strategy and a different LLM. The text within each bar displays the success rate, the number of programming tasks solved, and the total number of tasks. Rows are ordered according to the average success rates for both five attempts and one attempt across all tasks. Stars with different colors indicate the success rates of strategies that are significantly different (adjusted p‐value < 0.05) from those of the three strategies using GPT‐4 and Python 3. The p‐values were obtained using prop.test in R and adjusted for multiple testing using the BH procedure^[^ ^29^ ^]^ Sample sizes are indicated in the parentheses within the text.

**Figure** **2** shows the one‐attempt and five‐attempt success rates for different prompt strategies, programming languages, and tasks with varying difficulty levels for GPT‐4. The five‐attempt accuracy of the repeated prompt strategy with example cases is 86%, 58%, and 15% for easy, medium, and hard LeetCode tasks, respectively, which closely resemble the accuracy of the previous study^[^ ^1^ ^]^ and demonstrate the reproducibility of results across benchmark studies. For Python3, we found that the feedback CI prompt has the best overall performance, followed by the feedback prompt. Both strategies substantially outperform the multiway prompt and repeated prompt. Specifically, the feedback CI prompt increases the five‐attempt performance of the repeated prompt by 16% for easy tasks, 48% for medium tasks, and 120% for hard tasks. These results suggest that different prompt strategies substantially impact GPT‐4's coding ability, and choosing the optimal prompt strategy can substantially improve performance. With the feedback CI prompt, GPT‐4 is able to correctly solve all easy tasks and 86% of medium difficulty tasks with five attempts, showing that GPT‐4 can reliably generate code for tasks with moderate difficulty. In addition, the repeated prompt with example cases outperforms the repeated prompt without example cases, showing that GPT‐4 benefits from the reasoning provided by the example test cases in a way similar to chain‐of‐thoughts.^[^ ^26^ ^]^ Moreover, GPT‐4 shows comparable performance across different programming languages when using the same feedback prompt strategy. The variation in performance due to different programming languages is smaller than that caused by different prompt strategies.

### 2.4 Comparing Success Rates of Different LLMs

After identifying the impact of prompt strategy on the performance of LLMs, we compared the success rates of seven LLMs (Methods). For Gemini Ultra, Gemini Pro, Claude 2, GPT‐3.5, and Llama 2, we utilized the feedback prompt strategy, as these LLMs lack access to environments similar to GPT‐4's CI system. For Code Llama, we employed a repeated prompt strategy because it often failed to comprehend error messages and repeatedly gave the same responses in subsequent attempts as it did in the initial attempt.

Similar to GPT‐4, GPT‐3.5 successfully solves all programming tasks before September 2021, but its success rate drops considerably afterward. A substantial performance drop is not observed for other LLMs, as shown in Figure S5 (Supporting Information). It must be noted that LLMs other than GPT‐4 and GPT‐3.5 do not explicitly report their knowledge cutoffs, and their training data likely includes programming tasks collected at any time. Additionally, LLMs such as Gemini Ultra and Gemini Pro have direct access to the Internet. Thus, the performance of these LLMs reported in this study may be exaggerated compared to their performance on programming tasks whose solutions cannot be found in the training data or online. These results should be interpreted with caution.

Figure 2 compares the performance of various LLMs to GPT‐4, employing different prompt strategies. Despite the potential advantages of other LLMs discussed previously, GPT‐4 consistently outperforms all other LLMs, even when utilizing the same prompt strategy. For instance, the five‐attempt success rate for medium tasks by Gemini Ultra, the best‐performing LLM aside from GPT‐4, is 36% lower than GPT‐4's rate using the same prompt strategy. While most LLMs demonstrate comparable performances on easy tasks, their success rates vary substantially on medium and hard tasks. All LLMs, excluding GPT‐4, can solve at most half of the medium tasks and only 15% of the hard tasks within five attempts, which is substantially lower than the success rate of GPT‐4 with the optimal prompt strategy.

### 2.5 Comparing Coding Performance of LLMs and Human Programmers

We then compared the coding performance of LLMs and human participants in LeetCode and GeeksforGeeks contests. In these contests, participants are asked to solve several programming tasks in 1.5 h, and are ranked based on the tasks successfully solved, the number of attempts taken to solve the tasks, and the total amount of time spent. After the contest ends, the statistics and rankings of participants are published and can be easily queried. LeetCode and GeeksforGeeks enforce the fairness of their contests through plagiarism detection systems, disqualifying participants if plagiarism is detected. Thus, these contests provide a way to more rigorously benchmark the performance of LLMs with human programmers. Tables S1 and S2 (Supporting Information) provide a comprehensive compilation of the prompts used, the programming code generated by the LLMs under different prompt strategies, and the evaluation results in both LeetCode and GeeksforGeeks contests.

LLMs participated in the contests in a mock manner, with us serving as the interface between the LLMs and the contest websites. The best‐performing strategy, the Python3 feedback CI prompt, was used for GPT‐4. For other LLMs, the prompt strategies were the same as those in Figure 2. Each LLM was given a maximum of five attempts for each task. It is important to note that the contests do not impose a restriction on the number of attempts allowed. Thus, the performance of LLMs could potentially be further improved with more attempts. After each LLM completed the contest, we recorded its performance and followed the same competition rules to calculate the scores and obtain the ranking for each LLM (Experimental Section).

**Figure 3:**
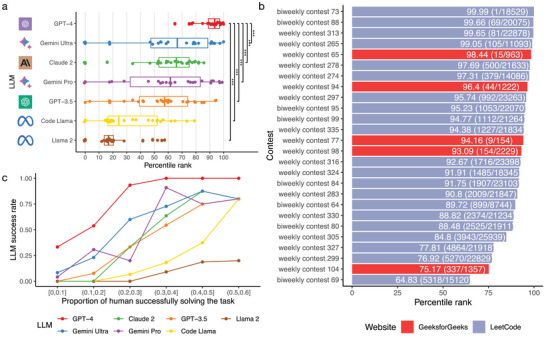
Comparing coding performances of LLMs and human programmers. a) Percentile rank (x‐axis) of LLMs (y‐axis) for LeetCode and GeeksforGeeks contests. Each dot is a coding contest. The Wilcoxon test was conducted to compare the percentile ranks of GPT‐4 with those of any other LLM. The p‐values were adjusted for multiple testing using the BH procedure.^[^ ^29^ ^]^ “***” indicates adjusted p‐value < 0.001. 26 contests were included. b) Percentile rank of GPT‐4 for LeetCode and GeeksforGeeks contests, colored differently. Each row represents a contest. Texts in the bar show the percentile rank of GPT‐4, the rank of GPT‐4, and total number of participants. c) LLM success rates (y‐axis) for LeetCode programming tasks categorized by proportion of human successfully solving the task (x‐axis). LLMs are represented by different colors.

**Figure** **3**a and Table S3 (Supporting Information) show the percentile rankings of all LLMs. GPT‐4 significantly outperforms other LLMs, consistent with the previous results shown in Figure 2. For most other LLMs, the percentile ranking is ≈60% for most coding contests, indicating that their coding capabilities are comparable to those of average human programmers. Figure 3b displays GPT‐4's percentile rankings, and the total number of participants for each coding contest. GPT‐4 demonstrates highly consistent performance in both LeetCode and GeeksforGeeks contests, indicating that the performance benchmark is reproducible. In 18 out of 26 contests (69%), GPT‐4 ranks above the 90th percentile. In 21 out of 26 contests (81%), GPT‐4 ranks above the 85th percentile. In three LeetCode contests with more than 18 000 participants, GPT‐4 ranks within the top 100. These results suggest that GPT‐4 possesses superior programming abilities compared to most human programmers participating in these contests, yet it still falls short of surpassing the most elite human programmers in most situations. It should be noted that the primary purpose of LeetCode and GeeksforGeeks is to help applicants prepare for interviews at technology companies. The interpretation of GPT‐4's results should be considered in the context that many human participants are likely students or professionals with moderate programming skills.

Figure 3c further compares the success rates of LLMs across programming tasks categorized by the proportion of human programmers who can successfully solve each task. More than 40% of human programmers fail the easiest task. The success rates of LLMs increase for tasks solvable by a larger percentage of human programmers. For tasks that more than 20% of human programmers can solve, GPT‐4's success rates exceed 90%. Even for tasks that less than 10% of human programmers can solve, GPT‐4 still achieves a success rate of 33%. These findings suggest that GPT‐4 can serve as a reliable assistant, enhancing the coding performance of most human programmers.

### 2.6 LLMs' Ability to Learn from Error Messages in Programming Code

Figure 2 shows that five‐attempt performances are substantially better than one‐attempt performances in most cases, suggesting that running additional attempts is needed to boost the success rate of programming tasks. However, the performance gain is different for different LLMs and prompt strategies. For GPT‐4, prompt strategies that rely on feedback messages benefit more from additional attempts. For the feedback CI prompt strategy, the five‐attempt success rates are 0.25 and 0.18 points higher than the one‐attempt success rates for medium and hard LeetCode tasks, respectively. These differences are reduced to 0.14 and 0.08, respectively, for the repeated prompt strategy.

**Figure 4:**
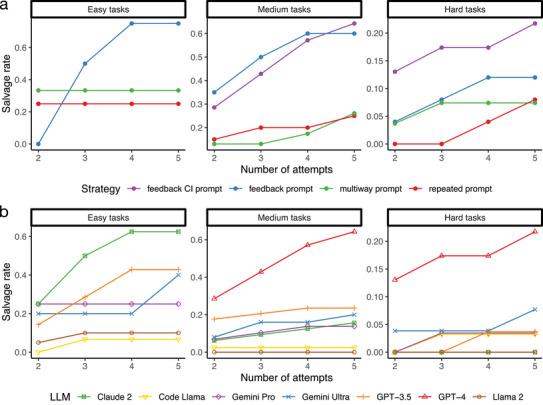
The salvage rates (y‐axis) increase with the number of attempts (x‐axis) for GPT‐4 using different prompt strategies a) and for different LLMs b). In (b), the feedback CI prompt strategy was used for GPT‐4, and the prompt strategies for other LLMs were the same as those in Figure 2.

To better understand this behavior of LLMs, **Figure** **4**a shows the salvage rate, defined as the success rate of subsequent attempts for programming tasks that failed the initial attempt, comparing GPT‐4 with different prompt strategies. For the repeated prompt and multiway prompt, running a second attempt salvages a considerable number of programming tasks that fail the first attempt, but running more than two attempts has a marginal effect on further increasing the success rate. In comparison, both the feedback prompt and the feedback CI prompt continue to benefit from additional attempts, eventually salvaging over 60% of tasks with easy and medium difficulties that failed the first attempt. The feedback CI prompt has the best overall salvage rate for hard tasks compared to other prompt strategies and is able to salvage over 20% of hard tasks that fail the first attempt. These results suggest that GPT‐4 is able to learn from the error messages and can iteratively fix its own coding errors with repeated attempts. In practical terms, running five attempts is generally sufficient, as the curves for both the feedback CI prompt and the feedback prompt tend to plateau beyond this point.

Figure 4b further compares the salvage rates of different LLMs. GPT‐4 exhibits the strongest performance in learning from previous errors, while Claude 2 and GPT‐3.5 also show considerable abilities, particularly in easy tasks. In contrast, other LLMs do not demonstrate a clear ability to learn from previous errors.

### 2.7 GPT‐4's Ability to Translate Across Programming Languages

**Figure 5:**
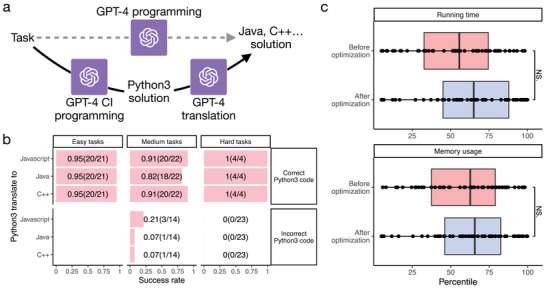
Evaluation of GPT‐4's abilities in translating across programming languages and the computational efficiency of the generated code. a) A schematic illustrates a proposed strategy to enhance the success rate of non‐Python3 programming languages using translation. b) The success rate of translating Python3 code, generated from one‐attempt feedback CI prompts, to other programming languages for tasks of varying difficulty levels. The top and bottom panels display results for when the original Python3 code is correct or incorrect, respectively. Since Python3 code generated by GPT‐4 is correct for all easy tasks, translation is not evaluated for easy tasks when the Python3 code is incorrect. c) Running time and memory usage percentile for GPT‐4 generated code compared to human programmers before and after optimization by GPT‐4. A higher percentile represents lower running time and memory usage, indicating better computational efficiency. Paired t‐tests were conducted to compare the running time and memory usage before and after optimization. The p‐values were adjusted for multiple testing using the BH procedure.^[^ ^29^ ^]^ “NS.” indicates not statistically significant. 53 pairs were included for each comparison.

Figure 2 also shows that the Python3 translation strategy outperforms the feedback prompt for Java, JavaScript, and C++, suggesting an alternative approach to improve the success rate of programming languages that cannot access GPT‐4's CI function (**Figure** **5**a). This is dependent on both the superior performance of the feedback CI prompt and GPT‐4's high accuracy in translating across programming languages.

Figure 5b and Table S4 (Experimental Section) show the success rate of different programming languages translated from Python3 by GPT‐4. The original programming task and the Python3 code output from a previous GPT‐4 query for solving the task were given to GPT‐4 when GPT‐4 was asked to perform the translation. When the original Python3 code is correct, GPT‐4 accurately translates it in almost all tasks. The success rate is almost identical across different target languages. Surprisingly, GPT‐4 is still able to generate code that correctly solves the programming task even when the original Python3 code is incorrect in some medium tasks. These results suggest that GPT‐4 can serve as a reliable tool to translate code across programming languages in most cases.

### 2.8 Computational Efficiency of Code Generated by GPT‐4

We recorded the running time and memory usage of code generated by GPT‐4 and found them to be comparable to those of human programmers when GPT‐4 was not specifically instructed to optimize computational efficiency (Figure 5c; Table S5, Supporting Information). We then asked GPT‐4 to optimize the computational efficiency of the code it had previously generated (Experimental Section). Both running time and memory usage improve slightly in this case (Figure 5c). These results suggest that the code generated by GPT‐4 is not specifically optimized for computational efficiency, and indicate that GPT‐4 has a limited capability in improving the computational efficiency of existing codes.

### 2.9 Assessing GPT‐4 on HackerRank Certification Tests

We also applied GPT‐4 with the feedback CI prompt and allowed up to five attempts to take the HackerRank certification tests, which cover a broader range of topics in computer programming compared to LeetCode and GeeksforGeeks problems. Thus, HackerRank serves as a complementary platform to assess LLMs' coding abilities in a real‐world coding environment.

**Figure 6:**
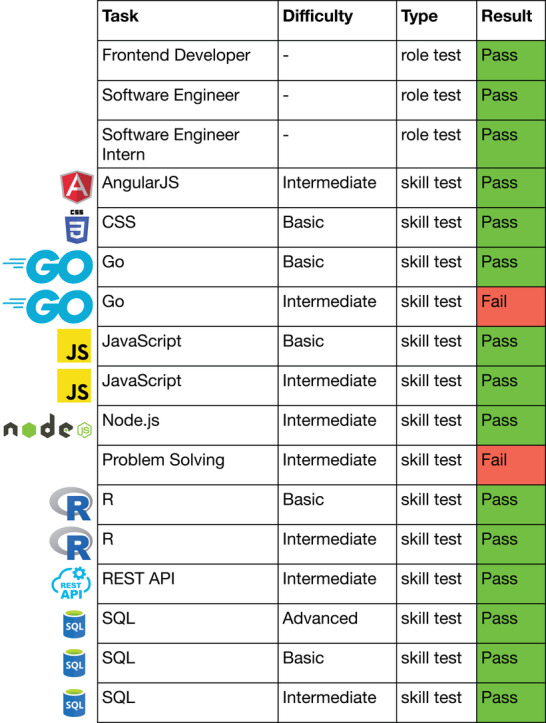
Results of GPT‐4 participating in HackerRank certification tests across various tasks, difficulty levels, and test types. Tests for which solutions published before September 2021 are not available online are included.

Unlike LeetCode and GeeksforGeeks, the publication dates of HackerRank coding tasks are unknown. Therefore, we conducted a comprehensive online search using Google for each task to determine if any solution had been posted before September 2021, the knowledge cutoff of GPT‐4. **Figure** **6** shows the results for tests where no solution posted before September 2021 could be found online. GPT‐4 passed all three role tests: front‐end developer, software engineer, and software engineer intern. Each role test consists of multiple coding tasks involving different programming languages and development frameworks. GPT‐4 also passed 12 out of 14 skill tests. The tests passed by GPT‐4 include applying development frameworks such as AngularJS and Node.js, front‐end design using CSS and JavaScript, applying R statistical software, calling Application Programming Interfaces (APIs) such as REST APIs, and performing database operations using SQL. These results suggest that GPT‐4 possesses versatile coding abilities and can excel in various roles. For comprehensiveness, Figure S6 (Supporting Information) shows the results for tests where solutions posted before September 2021 were available online.

### 2.10 A Customized Programming Assistant with GPTs

To facilitate program generation using GPT‐4, we created a programming assistant using the “GPTs” service provided by OpenAI (https://chatgpt.com/gpts). GPTs are customizable versions of GPT‐4 that allow users to tailor the model's behavior for specific tasks. We configured the programming assistant to generate code using the feedback CI prompt strategy. The assistant interacts with users to collect information such as the programming task description, example test cases, constraints, and code templates. Based on user instructions, the assistant can generate either the code or the prompt. Figure S7 (Supporting Information) illustrates an example of using the programming assistant. The programming assistant is available in the GPT store managed by OpenAI (https://chatgpt.com/g/g‐6745411e8b888191add9839a2cee2d3f‐programming‐assist).

## 3 Conclusion

In this study, we found that GPT‐4 is the best‐performing LLM in generating programming code. GPT‐4's performance is substantially influenced by various prompt strategies. The best prompt strategy utilizes GPT‐4's code interpreter function to test the validity of example test cases and benefits from the error messages from previous unsuccessful attempts. In LeetCode and GeeksforGeeks contests, GPT‐4 exhibits stronger coding performance than most human programmers, while other LLMs perform similarly to average human programmers. GPT‐4 demonstrates reliable coding performance across various programming languages and shows a strong ability to translate code between different programming languages. GPT‐4 also excels in tasks such as front‐end design, the application of development frameworks, API integration, and database operations.

These results suggest that GPT‐4 may serve as a reliable tool in generating programming code for practical applications. GPT‐4 may empower individuals without strong programming expertise to solve programming tasks of easy or medium difficulty. For those who have advanced programming expertise, GPT‐4 may share the workload, allowing human programmers to focus on more challenging tasks. Thus, GPT‐4 introduces a new paradigm in human‐AI collaboration, paving the way for a more efficient and inclusive process in generating programming code.

This study introduces several innovations to advance the understanding of LLM programming capabilities. It presents a novel framework that uses programming contests to fairly compare the coding performance of humans and LLMs, addressing biases present in previous task‐based evaluations. It also advances theoretical understanding by systematically comparing LLM performances under different prompt strategies, demonstrating their versatility across programming languages and validating their computational efficiency relative to human programmers. Furthermore, it highlights LLMs' capabilities in diverse programming tasks, provides practical guidance for optimal LLM usage, and offers actionable insights for integrating LLMs with human programmers to enhance efficiency, particularly through advanced prompting strategies like feedback CI prompts.

Programming with the aid of LLMs also brings new challenges. First, LLMs relies on well‐documented descriptions of programming tasks and comprehensive sets of test cases for validating the generated programming code. This reliance necessitates human involvement in determining the coding task, designing optimal prompts, and inputting those prompts, as well as additional training in crafting appropriate prompts, testing code, and iterating on it for more realistic tasks. Second, the use of commercial LLMs is not free of charge. For example, GPT‐4 and Claude have a monthly subscription fee of $20 for their online web version, and the cost increases linearly with the number of input and output tokens when using their API. The financial burden can become substantial and must be considered when LLMs are deployed to handle a large number of tasks. Third, information included in the prompt could be collected by the companies that develop and operate the LLM platforms, potentially leading to data privacy issues. Fourth, the use of LLMs raises several ethical considerations, including potential biases in model training and the responsible use of AI‐generated outputs. These concerns underscore the need for transparency in model development, rigorous validation processes, and the implementation of safeguards to mitigate unintended consequences and ensure equitable outcomes across diverse applications. Fifth, training and utilizing LLMs involves significant energy consumption, which can contribute to environmental challenges and exacerbate climate change. Addressing these concerns is crucial to ensuring the ethical and equitable deployment of LLMs in a programming environment.

This study has several limitations. First, this study evaluates only the abilities of LLMs to generate programming code, which is a subarea of software engineering. The abilities of LLMs to plan and manage projects, collaborate with team members, gain an in‐depth understanding of business logic, and perform many other tasks throughout the software development lifecycle have not been assessed in this study. Additionally, the coding tasks evaluated in this study may not fully replicate real‐world programming environments. Designing a more comprehensive and realistic framework for assessing coding performance is beyond the scope of this study but is an important avenue for future research. Second, despite a general understanding of the training data and procedures used to train commercial LLMs (e.g., the use of reinforcement learning from human feedback), the exact details are not publicly disclosed, as they often remain proprietary. This lack of transparency makes it difficult to assess the reliability and credibility of the information the model provides. Third, while we observed a correlation between the difficulty levels of LeetCode programming tasks and users' success rates (Figure S8, Supporting Information), the exact criteria used by LeetCode to define these difficulty levels remain unknown. In addition, although we collected certain demographic information about users visiting the LeetCode, GeeksforGeeks, and HackerRank websites from a third‐party source (Figure S9, Supporting Information), the specific details of users who participate in these programming contests are also unknown. While such information is largely proprietary, having access to these details could improve the interpretation of the conclusions drawn in this study.

## 4 Experimental Section

### Prompt Construction

Each LeetCode programming task was copied directly from the LeetCode website. The task includes the description of the problem (Figure S3a, Supporting Information), multiple example cases with inputs, desired outputs, and explanations (Figure S3b, Supporting Information), numerical constraints of parameters (Figure S3c, Supporting Information), and a code template consisting of several lines of code (Figure S3d, Supporting Information). The following sentence was added before the code template: “Code starts with:.” Each GeeksforGeeks programming task was manually transcribed from the GeeksforGeeks website, with the information organized in the same manner as the LeetCode tasks.

To construct the prompt for repeated prompt strategy with example test cases, the following sentence was added to the top of the full LeetCode programming task: “write Python3 code for the following question:.”

For the repeated prompt strategy without example test cases, the same prompts as the repeated prompt strategy with example test cases were used, except the example test cases were removed.

For the multiway prompt strategy, the following sentence was added to the top of the full LeetCode programming task: “write Python3 code for the following question using five different ways:.”

For the feedback prompt strategy, the first attempt uses the same prompt as the repeated prompt strategy, where “Python3” is replaced with “Java,” “Javascript,” or “C++” for other programming languages. For subsequent prompts, error messages (Figure S3e, Supporting Information) were directly copied and pasted from LeetCode to GPT‐4.

For the feedback CI prompt strategy, the initial prompt is identical to that of the feedback prompt, except that the sentence “Use a code interpreter to check the following examples:” is added above the example test cases. In subsequent attempts, if the error message from the previous attempt pertains to incorrect test cases, the new prompt will start with “Use code interpreter to check the following examples:,” followed by the example test cases from the original programming task and all incorrect test cases from previous failed attempts. If the error message is related to other issues, the error message is directly copied and pasted from LeetCode to GPT‐4.

For translating Python3 programming code into another programming language, the prompt is composed of the following components in order: “Convert the following Python3 code into Java”; “The Java Code starts with:”; a Java code template copied from LeetCode; “The Python3 code:”; the Python3 code generated previously by the GPT‐4 feedback CI prompt strategy for solving the programming task; “The original task was:”; and the prompt used in the Python3 repeated prompt strategy. “Java” is replaced with “Javascript” or “C++” for other programming languages.

To optimize the memory usage and running time of Python3 code, the following sentence was added above the Python3 code generated previously by the GPT‐4 feedback CI prompt strategy: “Improve the memory usage and running time of the following Python3 code.”

Prompts were constructed using similar approaches for GeeksforGeeks and HackerRank programming tasks.

### Query of GPT‐4

For GPT‐4, all programming tasks in this study were tested using the “gpt‐4‐0613” model. GPT‐4 queries were performed using the OpenAI Playground online web portal (https://platform.openai.com/playground).

For the multiway prompt strategy, GPT‐4 was queried once. For all other prompt strategies, GPT‐4 was queried up to five times.

The repeated prompt strategy and the feedback prompt strategy begin with the same initial prompt. For each task, the feedback prompt strategy was used first. The output from the first attempt of the feedback prompt strategy was then directly used as the first attempt's output for the repeated prompt strategy, rather than starting the latter from scratch. The repeated prompt strategy was then allowed up to four additional attempts. Therefore, the one‐attempt performances of the feedback and repeated prompt strategies are identical. This approach ensures that the comparison between these two strategies is not influenced by the randomness in the results of the first attempts.

### Query of Other LLMs

GPT‐3.5 queries were performed using the “gpt‐3.5‐turbo‐0613” model with the OpenAI Playground online web portal (https://platform.openai.com/playground).

Gemini Ultra 1.0 and Gemini Pro were accessed with the Gemini website (https://gemini.google.com/) from February 10 2024 to February 19 2024.

Claude 2 (Claude‐2‐100k), Llama 2 (Llama‐2‐70b), and Code Llama (Code‐Llama‐34b) were accessed with the Quora Poe app (https://poe.com/) from January 5 2024 to February 1 2024.

### Selection of Programming Tasks

For LeetCode, a list of all contests posted between January 2020 and September 2020 (one year before GPT‐4's knowledge cutoff in September 2021) was obtained. Four contests were randomly selected without replacement, with each contest having an equal probability of being chosen. Similarly, 21 contests posted between October 2021 and March 2023 were randomly selected using the same procedure.

For GeeksforGeeks, five contests posted between August 2022 and June 2023 were randomly selected using the same procedure.

All 24 HackerRank certification tests available on the HackerRank website as of November 2024 were evaluated in this study.

### Scoring and Ranking of Leetcode Contests

LeetCode assigns a numerical score to each of the four programming tasks in a LeetCode contest. The overall contest score is the sum of the scores for tasks successfully solved. To calculate the total time an LLM spent on a contest, the time for a complete LLM attempt is considered as 1 min, although in real practice, it often takes less than 1 min. A complete LLM attempt includes preparing the prompt by copying task information from LeetCode, querying the LLM, transferring the programming code from the LLM output to LeetCode, and running the evaluation on LeetCode. Per LeetCode contest rules, each failed attempt incurs a penalty time of 5 min. Therefore, if an LLM solves the task at the *k*th attempt (*k* ⩽ 5), the time the LLM spent on that task is *k* + (*k* − 1)*5 min. The overall time for a contest is the sum of the time spent on tasks successfully solved.

LeetCode ranks participants by their overall scores in descending order. For participants with tied scores, LeetCode ranks participants by their overall time spent in increasing order. The ranking list of human participants was manually queried by LeetCode and the ranking of an LLM was identified. If an LLM achieves an overall score of 0, its ranking is assigned as the mean ranking of all human participants who also scored 0.

### Scoring and Ranking of Geeksforgeeks Contests

GeeksforGeeks assigns a numerical score to each of the three programming tasks in a GeeksforGeeks contest. The overall contest score is the sum of the scores for problems successfully solved. Each contest problem's score incurs a 5% penalty for each wrong submission. For example, the score for a problem originally worth 40 points will be reduced to 38 points if there is one wrong submission.

GeeksforGeeks ranks participants according to their overall scores in descending order. In cases of tied scores, participants are further ranked based on the date and time of their last correct submission, with earlier submissions receiving higher rankings. However, GeeksforGeeks does not account for the time taken to complete the contest. Since there is no penalty for completion time, an LLM's rank was determined to be that of the best‐ranked human participant with the same overall score, assuming that all LLMs generate programming code faster than any human participant. GeeksforGeeks does not maintain records for participants with an overall score of 0. If an LLM achieves an overall score of 0, its ranking is determined by adding one to the total number of human participants who have positive overall scores.

### Evaluation of HackerRank Tests

GPT‐4's code was copied and pasted, generated for solving HackerRank programming tasks, onto the HackerRank website, which also evaluates the code. HackerRank then notifies users of the results, indicating whether a test was passed or failed, via email. No scoring or ranking was conducted locally.

### Implementation of the Customized Programming Assistant

The “GPTs” service provided by OpenAI was utilized to build the programming assistant. In the “Configure” window, the “Name” was set to “Programming Assist,” the “Description” was set to “An assistant for generating prompts for programming,” and the “Conversation starters” were set to “Generate code or prompts for programming tasks.” The capabilities of Web Search, DALL·E Image Generation, and Code Interpreter and Data Analysis were all enabled. The “Instructions” were set as follows:

Your task is to generate programming prompts and code. Strictly follow these steps to obtain information from users step‐by‐step. Do not ask for other types of information unless the user provides it spontaneously.

Determine Programming Language: Ask the user what programming language they would like to use.

Understand the Task: Ask the user to describe the programming task in detail.

Gather Example Test Cases: Request example test cases from the user, including Input data, Expected output, and Potential explanations for the results

Identify Constraints: Ask the user to specify any constraints or requirements for the solution (e.g., time complexity, libraries, or specific methodologies).

Code Template: Inquire if the user has a preferred code template to structure the solution.

Generate Code or Prompt: Finally, ask if the user wants to directly generated code, or a programming prompt for further use.

If the user chooses “Generate a Prompt,” use the following structure:

Write [Programming Language] code for the following question:

[Task Description]

For Python3, add: “Use a code interpreter to check the following examples:”

Example Test Cases: [List of cases with input, output, and explanations]

Constraints: [Any user‐provided constraints]

Code starts with: [Code template, if provided]

## Conflict of Interest

All authors declare no competing interests.

## Author Contributions

Z.J. and W.H. both conceived the study, conducted the analysis, and wrote the manuscript.

## Supporting information

Supporting Information

## Data Availability

All data generated or analyzed during this study are included in the supplementary tables.
