# Supplementary material for: Comparing Large Language Models and Human Programmers for Generating Programming Code
Source: Adv Sci (Weinh). 2024 Dec 30;12(8):2412279. doi: 10.1002/advs.202412279 (PMC11848527; doi:10.1002/advs.202412279)
Supplement: Supplementary file 1 — Supporting Information [file ADVS-12-2412279-s001.pdf]

ADVANCED SCIENCE
Open Access

Supporting Information

for *Adv. Sci.*, DOI 10.1002/advs.202412279

Comparing Large Language Models and Human Programmers for Generating Programming Code

Wenpin Hou and Zhicheng Ji**

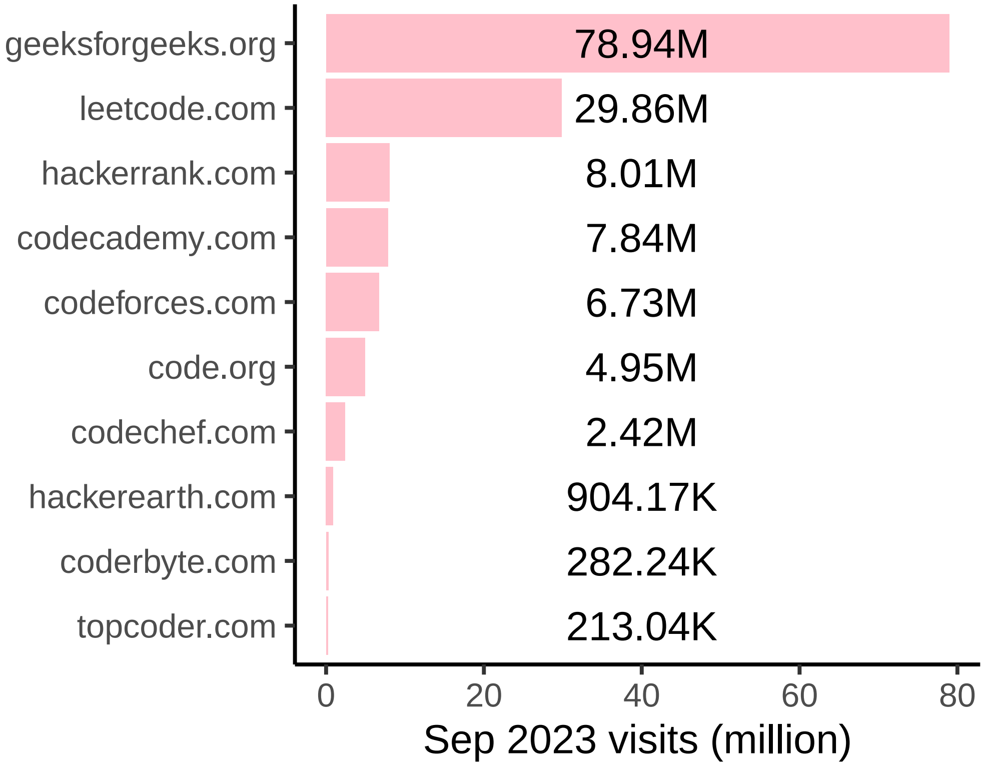

Supplementary Figure 1: Number of website visits in September 2023 for different platforms for practicing coding. The statistics were obtained from the Semrush website (`https://www.semrush.com/`).

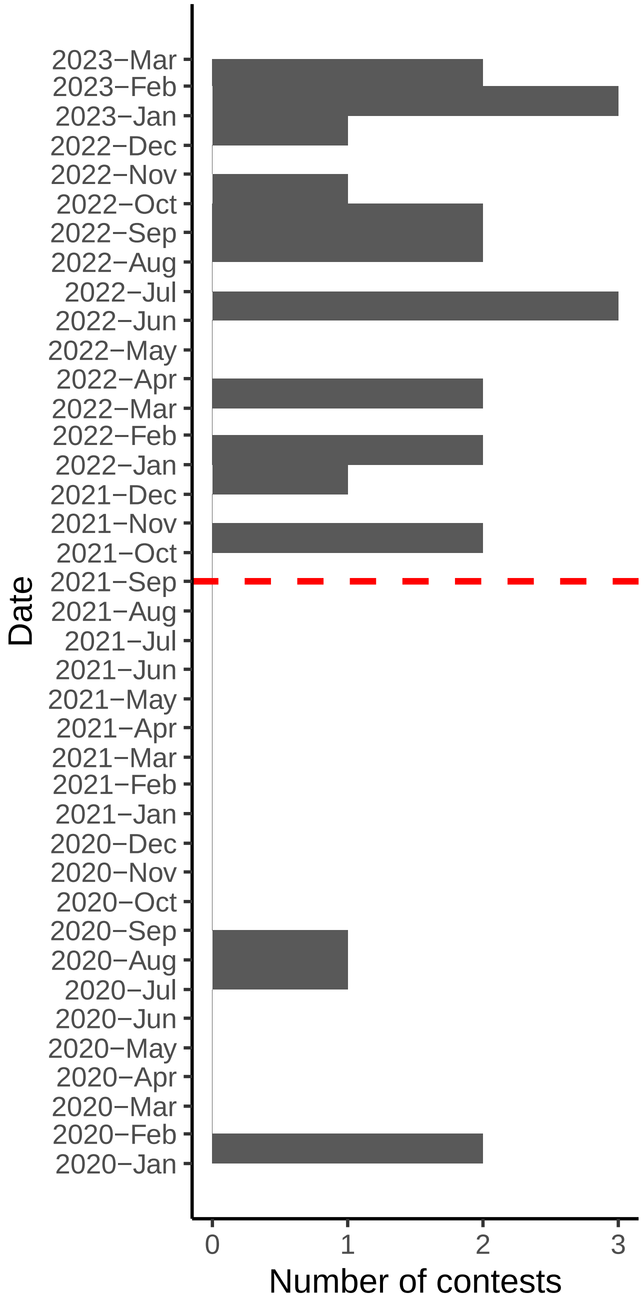

Supplementary Figure 2: Number of LeetCode contests published in different months and years.

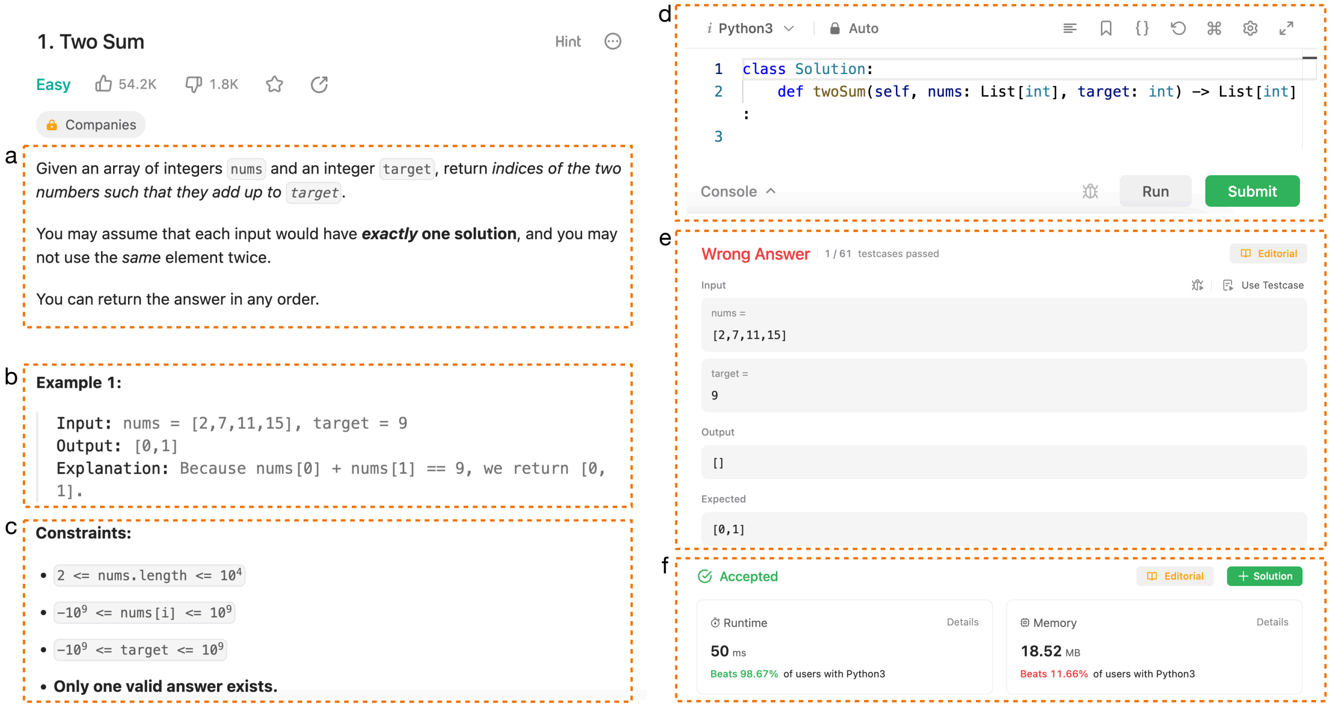

Supplementary Figure 3: Example screenshots of LeetCode programming tasks and evaluation results. **a**, Task description. **b**, Example test cases. **c**, Numerical constraints. **d**, Code template. **e**, Error message including a failed test case. **f**, Success message including running time and memory usage statistics.

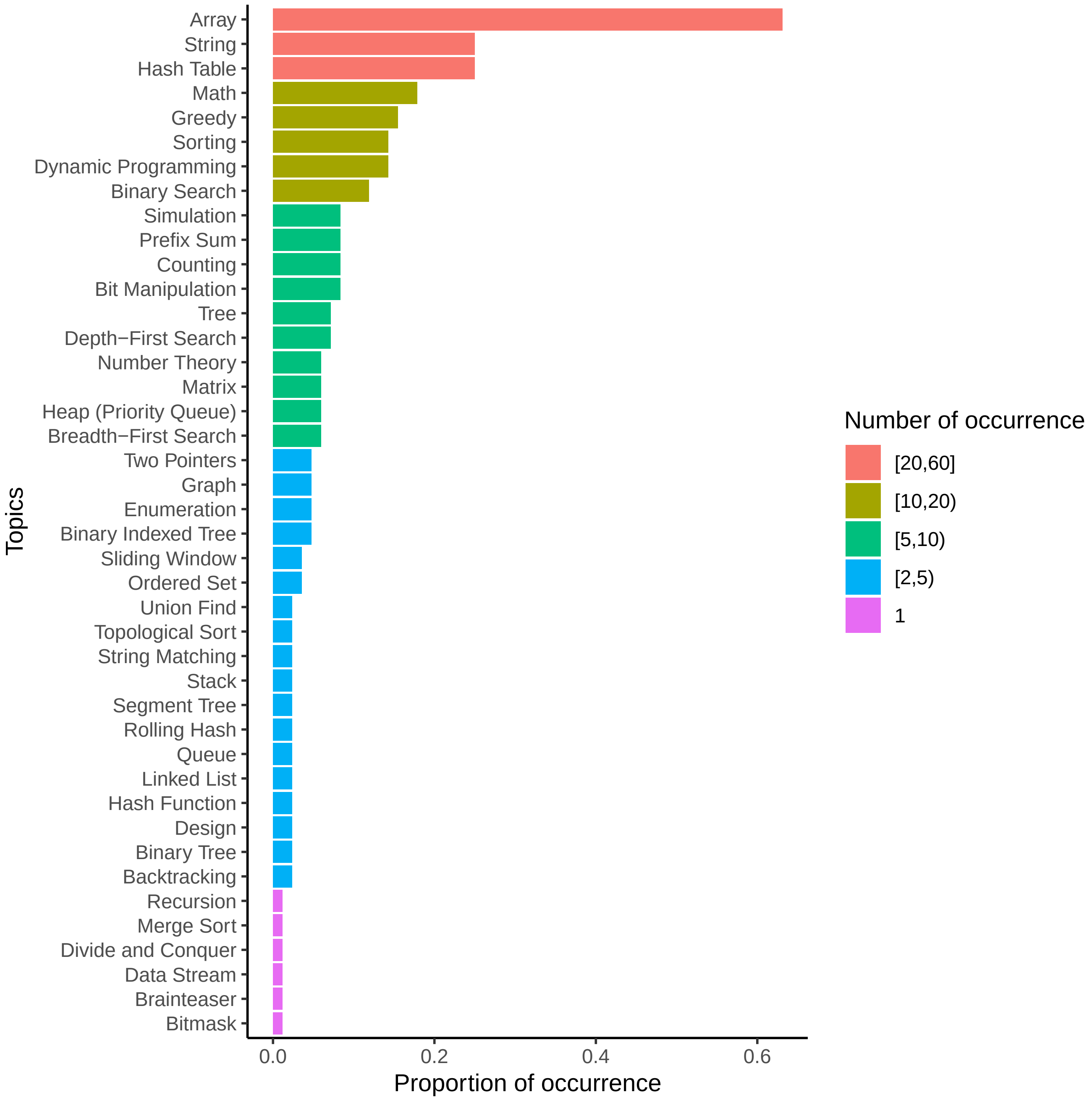

Supplementary Figure 4: Proportion of occurrences (x-axis) of different programming topics (y-axis) across all LeetCode programming tasks published after September 2021. The bars are colored by the number of occurrences.

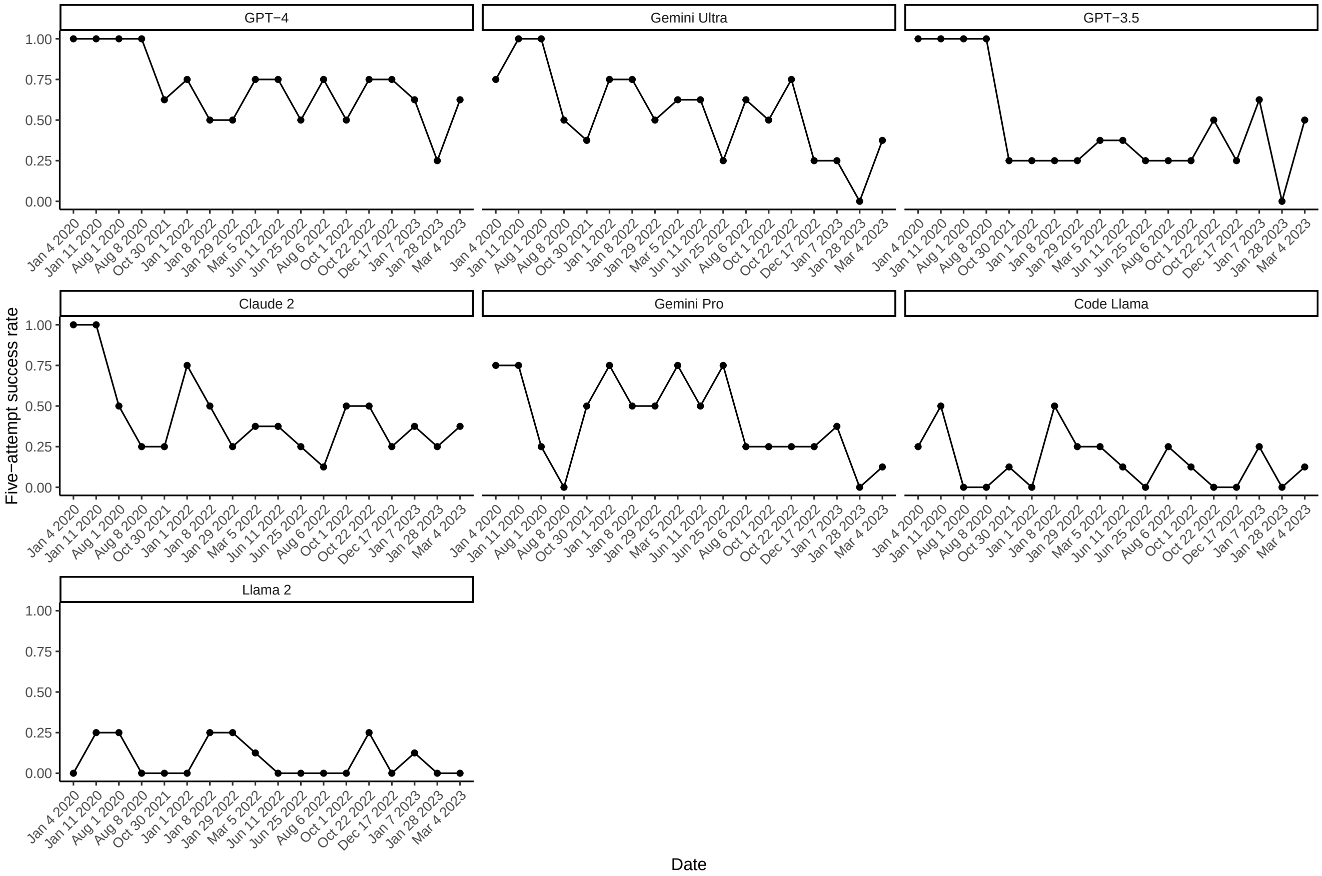

Supplementary Figure 5: Five-attempt success rates of different LLMs generating Python3 code for LeetCode contests published at different dates. For Code Llama, the repeated prompt strategy was used. For all other LLMs, the feedback prompt strategy was used.

Task	Difficulty	Type	Result
AngularJS	Basic	skill test	Pass
C#	Basic	skill test	Pass
Java	Basic	skill test	Pass
Node.js	Basic	skill test	Fail
Problem Solving	Basic	skill test	Pass
Python	Basic	skill test	Pass
React	Basic	skill test	Pass

Supplementary Figure 6: Results of GPT-4 participating in HackerRank certification tests across various tasks, difficulty levels, and test types. Tests for which solutions published before September 2021 are available online are included.

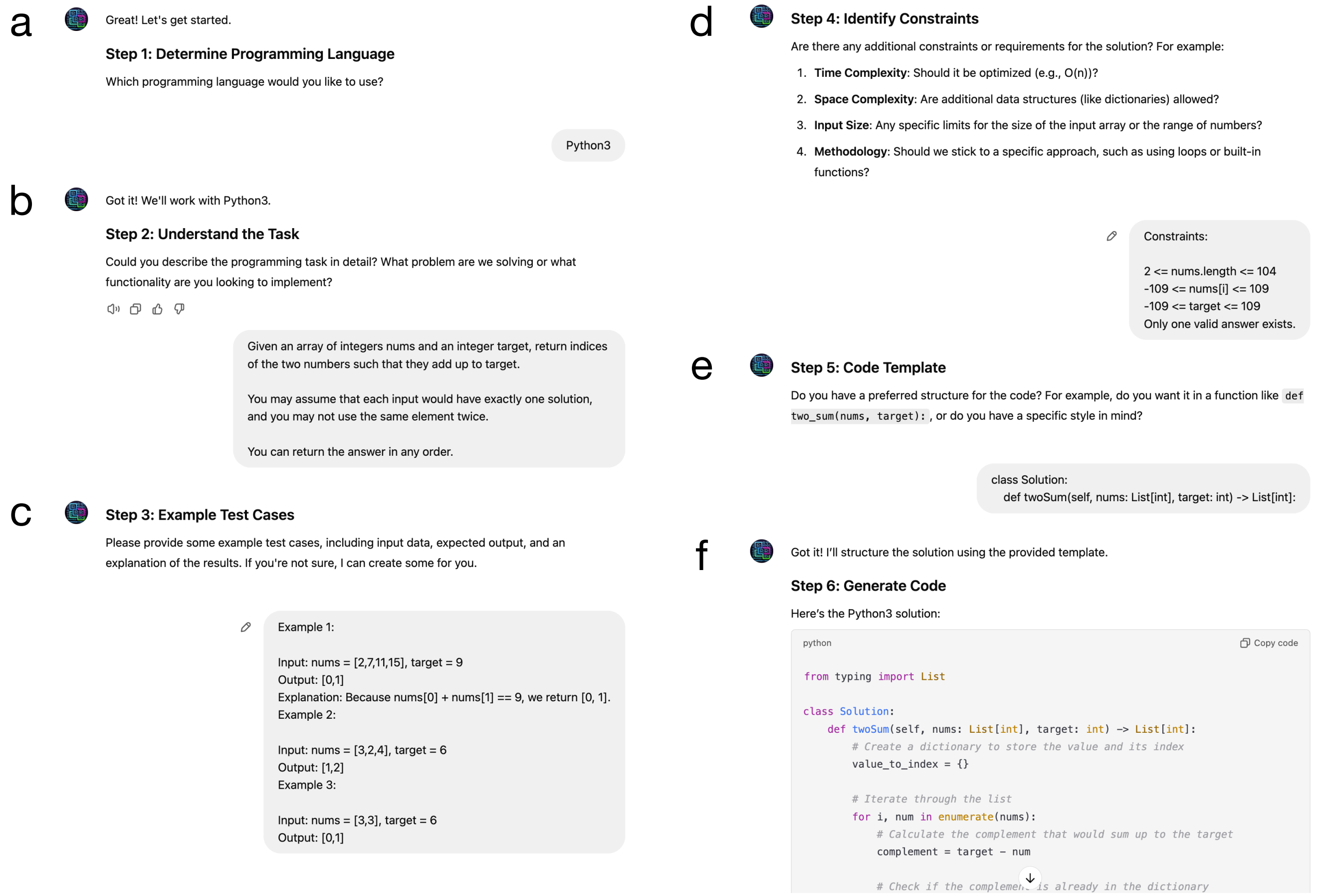

Supplementary Figure 7: An example of using the programming assistant developed based on GPTs. **a-e**, The user provides the programming language (**a**), details of the task (**b**), example test cases (**c**), constraints (**d**), and a code template (**e**). The programming assistant then generates code based on the user's input (**f**).

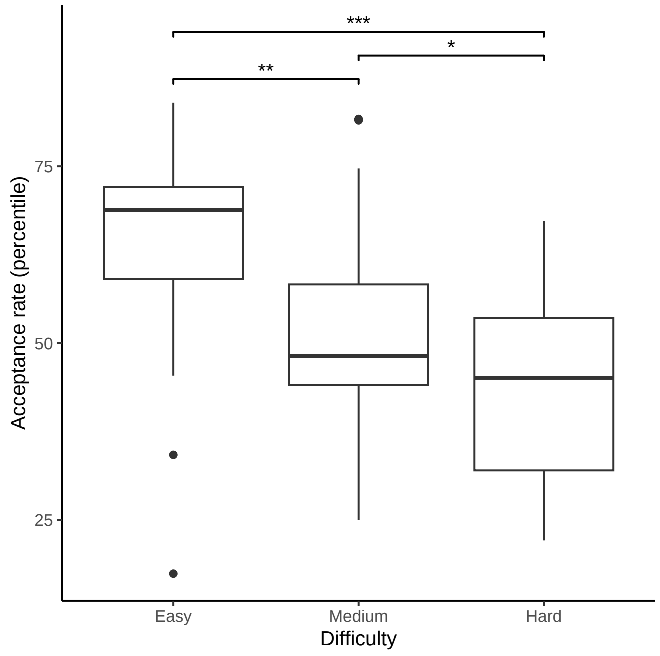

Supplementary Figure 8: Acceptance rates (y-axis) and difficulty levels of LeetCode programming tasks published after September 2021. Both pieces of information were directly obtained from the LeetCode website.

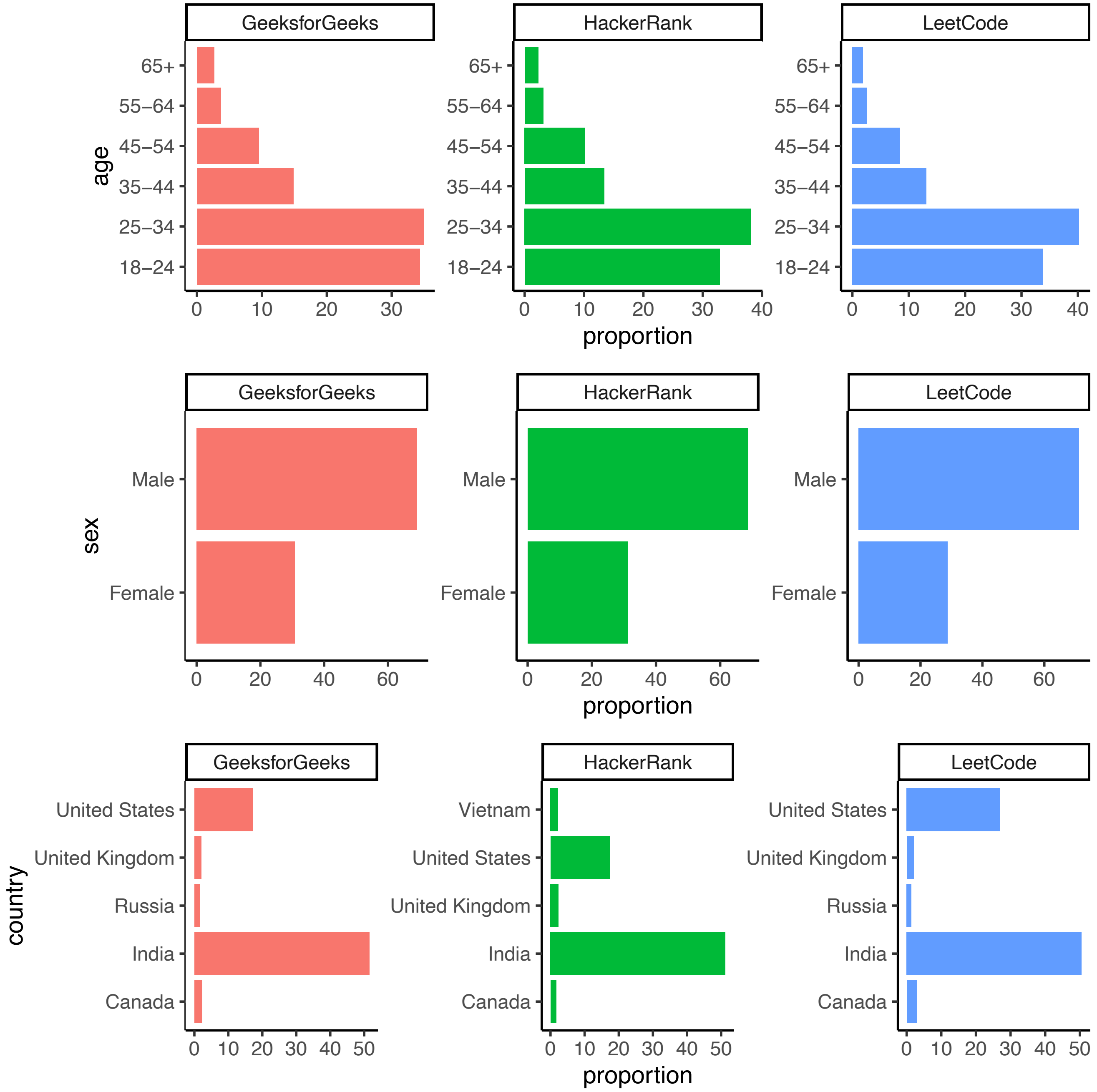

Supplementary Figure 9: Age, sex, and country distributions of visitors to the LeetCode, GeeksforGeeks, and HackerRank websites. This information was obtained from Similarweb.com in November 2024.
